# The extent of the stop coannihilation strip

**DOI:** 10.1140/epjc/s10052-014-2947-7

**Published:** 2014-07-09

**Authors:** John Ellis, Keith A. Olive, Jiaming Zheng

**Affiliations:** 1 Theoretical Particle Physics and Cosmology Group, Department of Physics, King’s College London, London, WC2R 2LS UK; 2 Theory Division, CERN, 1211 Geneva 23, Switzerland; 3 School of Physics and Astronomy, University of Minnesota, Minneapolis, MN 55455 USA; 4 William I. Fine Theoretical Physics Institute, School of Physics and Astronomy, University of Minnesota, Minneapolis, MN 55455 USA

## Abstract

Many supersymmetric models such as the constrained minimal supersymmetric extension of the Standard Model (CMSSM) feature a strip in parameter space where the lightest neutralino $\chi$ is identified as the lightest supersymmetric particle, the lighter stop squark ${\tilde{t}_1}$ is the next-to-lightest supersymmetric particle (NLSP), and the relic $\chi$ cold dark matter density is brought into the range allowed by astrophysics and cosmology by coannihilation with the lighter stop squark ${\tilde{t}_1}$ NLSP. We calculate the stop coannihilation strip in the CMSSM, incorporating Sommerfeld enhancement effects, and we explore the relevant phenomenological constraints and phenomenological signatures. In particular, we show that the ${\tilde{t}_1}$ may weigh several TeV, and its lifetime may be in the nanosecond range, features that are more general than the specific CMSSM scenarios that we study in this paper.

## Introduction

The non-appearance of supersymmetry during Run 1 of the LHC has given many theorists pause for thought. However, they should be encouraged by the fact that the Higgs boson has been discovered [1, 2] within the mass range predicted by simple supersymmetric models [3–15], and that its principal production and decay modes have occured at rates similar to those predicted for the Higgs boson of the Standard Model, also as predicted by simple supersymmetric models. The search for supersymmetry will continue during Run 2 of the LHC at higher energies and luminosities, which will have greatly extended physics reach compared to Run 1. It is important that this renewed experimental effort be matched by a thorough theoretical exploration of the different possible phenomenological signatures.

Many supersymmetric models, such as the constrained minimal supersymmetric extension of the Standard Model (CMSSM) [16–29], incorporate $R$-parity conservation, in which case the lightest supersymmetric particle (LSP) is stable and could provide astrophysical dark matter [30–39]. We assume here that the LSP is the lightest neutralino $\chi$ [40, 41]. There are several regions of the CMSSM parameter space where the relic $\chi$ density may fall within the range allowed by astrophysical and cosmological observations. Among the possibilities that have been most studied are the strip where stau-$\chi$ coannihilation is important [42–48], the funnel where there is rapid $\chi \chi$ annihilation via direct-channel heavy Higgs poles [16–19, 49–51], and the focus-point region where the $\chi$ acquires a significant Higgsino component [52–56]. The purpose of this paper is to pay closer attention to another possibility, namely the strip in the CMSSM parameter space where stop-$\chi$ coannihilation is important [57–63].

Generally speaking, the allowed parameter space of the CMSSM for any fixed values of $\tan \beta$ and $A_0/m_0$ may be viewed as a wedge in the $(m_{1/2}, m_0)$ plane. Low values of $m_0/m_{1/2}$ are excluded because there the LSP is the lighter stau slepton, which is charged and hence not a suitable dark matter candidate. The stau coannihilation strip runs along the boundary of this forbidden region [42–48]. High values of $m_0/m_{1/2}$ are also generically excluded, though for varying reasons. At low $A_0/m_0$, the reason is that no consistent electroweak vacuum can be found at large $m_0/m_{1/2}$, and close to the boundary of this forbidden region the Higgs superpotential mixing parameter $\mu$ becomes small, the Higgsino component of the $\chi$ gets enhanced, and one encounters the focus-point strip [52–56]. However, when $A_0/m_0$ is larger, the issue at large $m_0/m_{1/2}$ is that the LSP becomes the lighter stop squark ${\tilde{t}_1}$, which is also not a suitable dark matter candidate. Close to this boundary of the CMSSM wedge, the ${\tilde{t}_1}$ is the next-to-lightest supersymmetric particle, and the relic $\chi$ density may be brought into the cosmological range by ${\tilde{t}_1} \chi$ coannihilation [57–62]. The length of the ${\tilde{t}_1} \chi$ coannihilation strip is increased by Sommerfeld enhancements in some ${\tilde{t}_1} {\tilde{t}_1}^\star$ annihilation channels [64–69], which we include in our analysis.

In this paper we study the extent to which portions of this ${\tilde{t}_1} \chi$ strip may be compatible with experimental and phenomenological constraints as well as the cosmological dark matter density, paying particular attention to the constraint imposed by the LHC measurement of the mass of the Higgs boson. Other things being equal, the measurement $m_\mathrm{H} = 125.9 \pm 0.4$ GeV tends to favour larger values of $A_0$ such as those featuring a ${\tilde{t}_1} \chi$ coannihilation strip, reinforcing our interest in this region of the CMSSM parameter space [37–39, 70–84]. We use FeynHiggs 2.10.0 to calculate the lightest supersymmetric Higgs mass and to estimate uncertainties in this calculation [85]. We find that the stop coannihilation strip may extend up to $m_{1/2} \simeq 13000$ GeV, corresponding to $m_\chi = m_{\tilde{t}_1} \simeq 6500$ GeV, that the end-point of the stop coannihilation strip may be compatible with the LHC measurement of $m_\mathrm{H}$ for $\tan \beta = 40$ or large $A_0/m_0 = 5.0$ within the FeynHiggs 2.10.0 uncertainty, and that the stop lifetime may extend into the nanosecond range.

The layout of this paper is as follows. In Sect. 2 we review relevant general features of the CMSSM, setting the ${\tilde{t}_1} \chi$ coannihilation strip in context and describing our treatment of Sommerfeld enhancement effects. In Sect. 3 we study the possible extent of this strip and the allowed range of the ${\tilde{t}_1}$ mass. Although our specific numerical studies are the framework of the CMSSM, we emphasise that our general conclusions have broader validity. In Sect. 4 we discuss ${\tilde{t}_1}$ decay signatures, which are also not specific to the CMSSM, and in Sect. 5 we summarise our conclusions.

## Anatomy of the stop coannihilation strip

We work in the framework of the CP-conserving CMSSM, in which the soft supersymmetry-breaking parameters $m_{1/2}, m_0$ and $A_0$ are assumed to be real and universal at the GUT scale. We treat $\tan \beta$ as another free parameter and use the renormalisation-group equations (RGEs) and the electroweak vacuum conditions to determine the Higgs superpotential mixing parameter $\mu$ and the corresponding soft supersymmetry-breaking parameter $B$ (or, equivalently, the pseudoscalar Higgs mass $M_A$). We concentrate in the following on the choices $\mu > 0$ and $A_0 > 0$.

### Sommerfeld effect

We evaluate the dark matter density in the regions of the stop coannihilation strips including the Sommerfeld effect, which may enhance the annihilation rates at low velocities, and which is particularly relevant for strongly interacting particles such as the stop squark. As we discuss in more detail below, the general effect of including the Sommerfeld factors is to increase substantially the length of the stop coannihilation strip.

In general, the Sommerfeld effect modifies s-wave cross sections by factors [64]

1 $$\begin{aligned} F(s) \equiv \frac{- \pi s}{1 - \mathrm{e}^{\pi s}} : \; s \; \equiv \; \frac{\alpha }{\beta } \, , \end{aligned}$$

where $\beta$ is the annihilating particle velocity and $\alpha$ is the coefficient of a Coulomb-like potential whose sign is chosen so that $\alpha < 0$ corresponds to attraction. In the case of annihilating particles with strong interactions, the Coulomb-like potential may be written as [86–88]

2 $$\begin{aligned} V = \frac{\alpha _3}{2 r} \left[ C_f - C_i - C_i^\prime \right] \, , \end{aligned}$$

where $\alpha _3$ is the strong coupling strength at the appropriate scale, $C_i$ and $C_i^\prime$ are the quadratic Casimir coefficients of the annihilating coloured particles, and $C_f$ is the quadratic Casimir coefficient of a specific final-state colour representation.^1^ In our case, we always have $C_i = C_i^\prime = C_3 = 4/3$. In ${\tilde{t}_1}- {\tilde{t}_1}^{\star }$ annihilations the possible s-channel states are singlets with $C_1 = 0$ and octets with $C_8 = 3$, whereas in ${\tilde{t}_1}- {\tilde{t}_1}$ annihilations Bose symmetry implies that the only possible final colour state is a sextet with $C_6 = 10/3$. The factors in the square parentheses $[ ... ]$ for the singlet, octet and sextet final states are therefore $-8/3, + 1/3$ and $+ 2/3$, respectively, corresponding to $\alpha = - 4 \alpha _3/3, \alpha _3/6$ and $\alpha _3/3$, respectively. Only the singlet final state exhibits a Sommerfeld enhancement: s-wave annihilations in the other two colour states actually exhibit suppressions.

We implement the Sommerfeld effects in the SSARD code [89] for calculating the relic dark matter density, which is based on a non-relativistic expansion for annihilation cross sections:

3 $$\begin{aligned} \langle \sigma v \rangle = a + b x + \cdots \, , \end{aligned}$$

where $\langle ... \rangle$ denotes an average over the thermal distributions of the annihilating particles, the coefficient $a$ represents the contribution of the s-wave cross section, $x \equiv T/m$, and the dots represent terms of higher order in $x$. When $\alpha < 0$ in (1), as in the singlet final state discussed above, the leading term in (3) acquires a singularity

4 $$\begin{aligned} a \rightarrow a \frac{\sqrt{2 \pi }}{x} + \cdots \, , \end{aligned}$$

where the dots again represent terms of higher order in $x$. The Sommerfeld correction to the annihilation cross section that we include is parametrically enhanced by a factor $1/x$ close to threshold, cf. our Eq. (4). Going beyond this term to include non-enhanced corrections would require a complete calculation of $\mathcal{O}(\alpha _s)$ corrections, which lies far beyond the scope of this paper.

Along the stop coannihilation strip, the dominant ${\tilde{t}_1} - {\tilde{t}_1}^\star$ s-wave annihilation cross sections are typically those into colour-singlet pairs of Higgs bosons ($\sim$60–70 % in the CMSSM before incorporating the Sommerfeld effect) and into gluon pairs ($\sim$20–30 %), which are a mixture of 2/7 colour-singlet and 5/7 colour-octet final states, followed by the colour-octet $Z$ + gluon final state ($\sim$5 % in the CMSSM). We have implemented the Sommerfeld effects for these ${\tilde{t}_1} - {\tilde{t}_1}^\star$ final states, and also for ${\tilde{t}_1} - {\tilde{t}_1} \rightarrow t + t$ annihilations, whose s-wave annihilation cross section $\sim$5 % of the total ${\tilde{t}_1}- {\tilde{t}_1}^\star$ s-wave annihilation cross section before including the Sommerfeld effect.

We emphasise that the Sommerfeld factors in different channels depend only on the final states and are independent of the specific CMSSM scenario that we study. We also emphasise that many other supersymmetric models feature the same suite of final states in stop–neutralino coannihilation. Moreover, some of the couplings to these final states are universal, e.g., ${\tilde{t}_1}- {\tilde{t}_1}^\star$ annihilations to gluon pairs mediated by crossed-channel ${\tilde{t}_1}$ exchange and direct-channel gluon exchange. The similarities imply that results resembling ours would hold in many related supersymmetric models.^2^

### The end-point of the stop coannihilation strip

As we shall also see, there are differences in the lengths of the stop coannihilation strips for different values of the model parameters. Looking at the dominant ${\tilde{t}_1} - {\tilde{t}_1}^\star$ annihilation mechanisms, it is clear that the matrix elements for annihilations to some final states are universal, e.g., to gluon pairs. However, the dominant ${\tilde{t}_1} - {\tilde{t}_1}^\star$ annihilations to pairs of Higgs bosons are model dependent. The dominant contributions to ${\tilde{t}_1} - {\tilde{t}_1}^\star \rightarrow h + h$ annihilation, in the notation of the appendix in [61], are $\mathrm I \times \mathrm I ,\mathrm II \times \mathrm II ,\mathrm I \times \mathrm II ,\mathrm I \times \mathrm III$ and $\mathrm II \times \mathrm III$ with $i=2$, corresponding to $t-$ and $u$-channel exchanges of the heavier stop ${\tilde{t}_2}$, the exchange of the lighter stop exchange being suppressed by $\sin \theta _t$, where $\theta _t$ is the ${\tilde{t}_1} - {\tilde{t}_2}$ mixing angle. The ${\tilde{t}_1} - {\tilde{t}_2}^{\star } - h$ coupling takes the form

5 $$\begin{aligned} C_{\tilde{t}_1-\tilde{t}_2-h} \; \sim \; \frac{\mu \sin \alpha -A_t\cos \alpha }{2m_\mathrm{W}\sin \beta }\cos 2\theta _t \, , \end{aligned}$$

which depends on $A_t$, $\sin \beta$, the Higgs mixing angle $\alpha$ and $\mu$, as well as $\theta _t$, and the annihilation cross section also depends on $m_{\tilde{t}_2}$. The ${\tilde{t}_1} - {\tilde{t}_1}^\star \rightarrow h + h$ annihilation rate is therefore model dependent, depending primarily on the combination $C_{\tilde{t}_1-\tilde{t}_2-h}/m_{\tilde{t}_2}$, which causes $m_\chi$ at the tip of the stop coannihilation strip to vary as we see later.

## Representative parameter planes in the CMSSM

### $(m_{1/2}, m_0)$ Planes

We display in Fig. 1 some representative CMSSM $(m_{1/2}, m_0)$ planes for fixed $\tan \beta = 20$, $\mu > 0$ and different values of $A_0/m_0$ that illustrate the interplay of the various theoretical, phenomenological, experimental and cosmological constraints.^3^ In each panel, any region that does not have a neutral, weakly interacting LSP is shaded brown. Typically there are two such regions which appear as triangular wedges. The wedge in the upper left of the $(m_{1/2}, m_0)$ plane contains a stop LSP or tachyonic stop, and the wedge in the lower right of the plane contains a stau LSP or tachyonic stau. The dark blue strips running near the boundaries of these regions have a relic LSP density within the range of the cold dark matter density indicated by astrophysics and cosmology [91]^4^: that near the boundary of the upper left wedge is due to stop coannihilation, and that near the boundary of the lower right wedge is due to stau coannhilation. As we discuss later, the stop coannihilation strips typically extend to much larger values of $m_{1/2}$ than the stau coannhilation strips, indeed to much larger values of $m_{1/2}$ than those displayed in Fig. 1, reaching as far as 7000–13000 GeV in the models studied. The green shaded regions are incompatible with the experimental measurement of $b \rightarrow s \gamma$ decay [92], and the green solid lines are 95 % CL constraints from the measured rate of $B_s \rightarrow \mu ^+ \mu ^-$ decay [93–95]. The solid purple lines show the constraint from the absence of $/ E_T$ events^5^ at the LHC at 8 TeV [97], and the red dot-dashed lines are contours of $m_\mathrm{H}$ calculated using FeynHiggs 2.10.0, which have a typical uncertainty $\pm$3 GeV for fixed input values of $m_{1/2}, m_0, \tan \beta$ and $A_0$ [85, 98–101]. We note that the multiple RGE solutions found in [102, 103] appear in regions of parameter space with either $\mu < 0$ and/or small $A_0 < m_0$ (mostly $A_0 = 0$)—whereas the stop coannihilation strips we study appear for $\mu > 0$ and $A_0/m_0 \ge 2$.

**Fig. 2:**
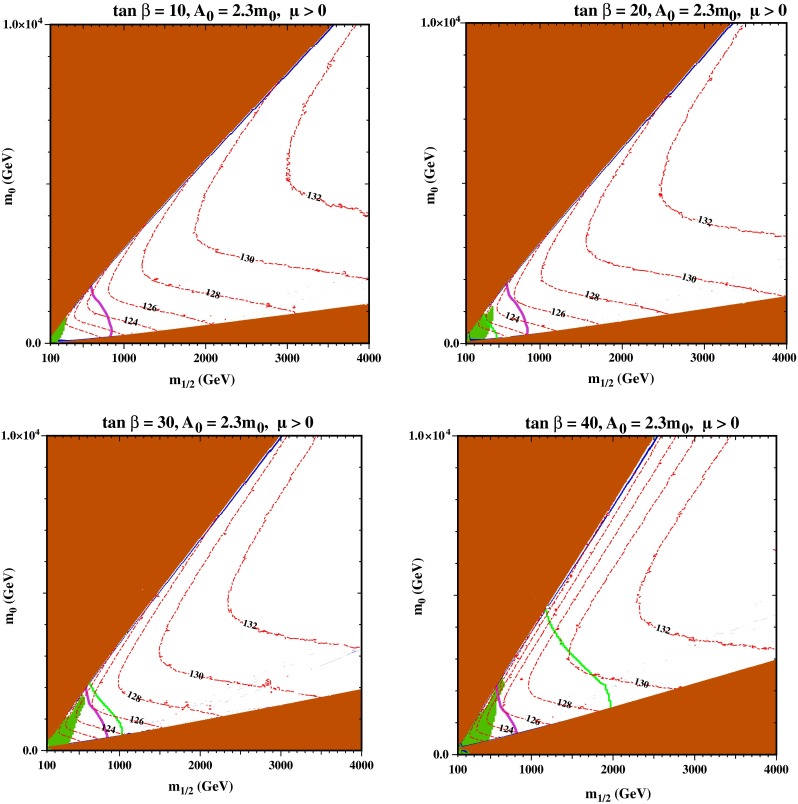
As Fig. 1, displaying the allowed regions in the $(m_{1/2}, m_0)$ planes for $A_0 = 2.3 \, m_0$ and $\tan \beta = 10$ (*upper left*), $\tan \beta = 20$ (*upper right*), $\tan \beta = 30$ (*lower left*) and $\tan \beta = 40$ (*lower right*). The *line styles* and *shadings* are described in the text

In general, we identify stop coannihilation strips in CMSSM $(m_{1/2}, m_0)$ planes for $2.1 \, m_0 \lesssim A_0 \lesssim 5.5 \, m_0$, and the panels in Fig. 1 have been chosen to represent the range of possibilities for $\tan \beta = 20$. The angle subtended by the (brown) stop LSP wedge increases with $A_0/m_0$, and this wedge meets the (brown) stau LSP wedge and closes the intermediate (unshaded) neutralino LSP wedge for $A_0 \gtrsim 5.5 \, m_0$.^6^ Each of the panels of Fig. 1 also features a stau coannihilation strip running close to the boundary of the stau LSP wedge, which extends to $m_{1/2} \sim 1000$ GeV corresponding to $m_\chi \sim 400$ GeV.

Along these strips, the LHC $/ E_T$ constraint excludes $m_{1/2} < 800$ GeV, but the excluded range of $m_{1/2}$ is reduced for the larger values of $m_0$ along the stop coannihilation strip. For the planes shown in Fig. 1, the stop strip extends far beyond the range of $m_{1/2}$ shown (see Sect. 4 below for more discussion as regards the end-point of the stop strips). However, depending on the ratio, $A_0/m_0$, the strip may conflicted with the measured value of the Higgs mass. For example, for $A_0/m_0 = 2.2$, the strip crosses $m_\mathrm{H} = 128$ GeV at $m_{1/2} \simeq 1100$ GeV. As $A_0/m_0$ is increased, the Higgs mass rapidly decreases along the strip. When $A_0/m_0 = 2.5$, the strip crosses $m_\mathrm{H} = 128$ GeV at $m_{1/2} \simeq 2600$ GeV and $m_{1/2} \gtrsim 1100$ GeV for $m_\mathrm{H} > 124$ GeV. For $A_0/m_0 = 3.0$, $m_{1/2} \gtrsim 2200$ GeV for $m_\mathrm{H} > 124$ GeV and the strip is allowed to extend to much higher $m_{1/2}$ than shown in the figure. For $A_0/m_0 = 5.0$, only the far end of the strip at large $m_{1/2} \gtrsim 4$ TeV is allowed. We return later to the impact of the LHC constraint on $m_\mathrm{H}$ and other phenomenological constraints on the stop coannihilation strip.

Figure 2 displays the sensitivity of the stop coannihilation strip to the choice of $\tan \beta$ for the representative choice $A_0 = 2.3 \, m_0$. Here we see that the opening angle of the stop LSP wedge is rather insensitive to $\tan \beta$, that of the stau coannihilation strip being more sensitive. Also, we recall that studies indicate that the LHC $/ E_T$ constraint is essentially independent of $\tan \beta$. On the other hand, the impacts of the $b \rightarrow s \gamma$ and $B_s \rightarrow \mu ^+ \mu ^-$ constraints increase with $\tan \beta$. They only ever exclude a fraction of the stop coannihilation strip, but the $B_s \rightarrow \mu ^+ \mu ^-$ constraint does exclude the entire stau coannihilation strip for $\tan \beta = 40$. The $m_\mathrm{H}$ contours calculated using FeynHiggs 2.10.0 are quite similar for $\tan \beta = 10, 20$ and 30. However, we find smaller values of $m_\mathrm{H}$ for $\tan \beta = 40$, a feature whose implications we discuss in more detail later.

### $(\tan \beta , A_0)$ Planes

**Fig. 3:**
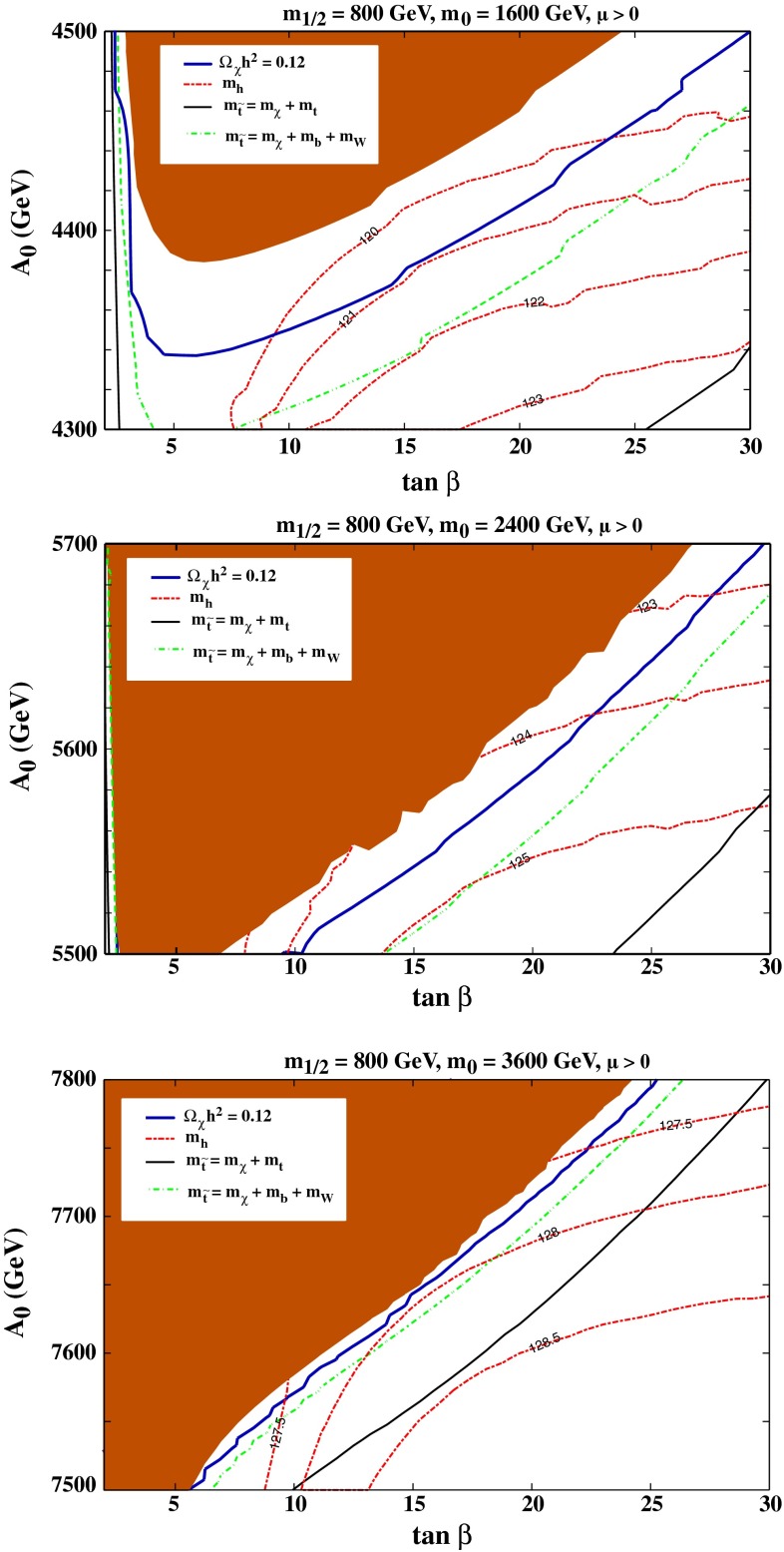
The CMSSM $(\tan \beta , A_0)$ planes for $(m_{1/2}, m_0) = (800, 1600/2400/3600)$ GeV in the *top/middle/bottom panels*, respectively. The (*brown*) *shaded* is excluded because $m_{\tilde{t}_1} < m_\chi$. Also shown are the contours $m_{\tilde{t}_1} = m_\chi + m_b + m_\mathrm{W}$ (*green, dash-dotted*) and $m_{\tilde{t}_1} = m_\chi + m_t$ (*black, solid*). The *solid blue line* is the strip where $\Omega _\chi h^2 = 0.12$ and the *red dash-dotted lines* are contours of $m_\mathrm{H}$ calculated with FeynHiggs 2.10.0

In view of the dependences of the stop coannihilation strips on the values of $\tan \beta$ and $A_0$, we display in Fig. 3 examples of $(\tan \beta , A_0)$ planes in the CMSSM for fixed $m_{1/2}$ and $m_0$. In the (brown) shaded region at the top of each panel, the ${\tilde{t}_1}$ is lighter than the $\chi$, so there is no weakly interacting neutral dark matter. Running below this boundary, the solid (blue) line is the contour where $\Omega _\chi h^2 = 0.12$. The other roughly parallel contours are $m_{\tilde{t}_1} = m_\chi + m_b + m_\mathrm{W}$ (green, dash-dotted) and $m_{\tilde{t}_1} = m_\chi + m_t$ (black, solid). Finally, the red dash-dotted lines are contours of $m_\mathrm{H}$ calculated using FeynHiggs 2.10.0. In each panel, we see that the calculated value of $m_\mathrm{H}$ increases with increasing $\tan \beta$ and decreases with increasing $A_0$, and comparing the panels for $m_0 = 1600$ GeV (top), 2400 GeV (middle) and 3600 GeV (bottom) we see that $m_\mathrm{H}$ also increases with $m_0$.

We see in the top panel of Fig. 3 for the combination $(m_{1/2}, m_0) = (800, 1600)$ GeV that $m_\mathrm{H} < 121$ GeV along the whole $\Omega _\chi h^2 = 0.12$ contour, so the LHC Higgs mass measurement rules out this combination of $m_{1/2}$ and $m_0$ for any value of $\tan \beta$ and $A_0$. On the other hand, we see in the middle panel for $(m_{1/2}, m_0) = (800, 2400)$ GeV that $m_\mathrm{H}> 122.5$ GeV (and hence is compatible with the measured value of $m_\mathrm{H}$ after allowing for the theoretical uncertainty $\sim$3 GeV in the FeynHiggs 2.10.0 calculation) along all of the displayed portion of the dark matter contour extending from $(\tan \beta , A_0) = (10, 5500)$ to $(28, 5700~\mathrm{GeV})$, corresponding to $A_0/m_0 \sim 2.4$. Finally, in the bottom panel of Fig. 3 we see that along all of the displayed portion of the dark matter contour extending from $(\tan \beta , A_0) = (6, 7500)$ to $(25, 7800~\mathrm{GeV})$ corresponding to $A_0/m_0 \sim 2.2$ we have $127 < m_\mathrm{H} < 128$ GeV, which is also compatible with the experimental measurement within the estimated theoretical uncertainties.^7^

Figure 4 displays analogous $(\tan \beta , A_0)$ planes for $(m_{1/2}, m_0) = (1200, 2400/3000/3600)$ GeV in the top/middle/bottom panels, respectively. We see in the top panel that $m_\mathrm{H}$ is compatible with the experimental value within the estimated theoretical uncertainty of $\sim$3 GeV only for $\tan \beta \sim 15$, where FeynHiggs 2.10.0 yields a nominal value $m_\mathrm{H} \simeq 122.5$ GeV. On the other hand, we see in the middle panel, where $m_0$ is increased to 3000 GeV, that LHC-compatible values of $m_\mathrm{H}$ are found for all values of $\tan \beta \in (5, 27)$, and the same holds true in the bottom panel, where $m_0 = 3600$ GeV. Value of $A_0/m_0$ in the displayed regions of the stop coannihilation strips range $\sim$2.3 to $\sim$2.7.

### $(m_{1/2}, A_0)$ Planes

Figure 5 displays some $(m_{1/2}, A_0)$ planes for fixed $(\tan \beta , m_0) \!=\! (15, 2400/3000/3600)$ GeV in the top/middle/bottom panels, showing the same mass and relic density contours as in the previous figures. In each of the three panels, we see that $m_\mathrm{H}$ decreases as we move along the strip to higher $m_{1/2}$. In the top panel, $m_\mathrm{H}$ falls below 123 GeV at $m_{1/2} \sim 1100$ GeV and lower values of $m_{1/2}$ are preferred. Since the relic density and Higgs mass contours are nearly parallel, in each panel of the lower two panels, we find LHC-compatible values of $m_\mathrm{H}$ along all of the displayed portion of the relic density contour from $m_{1/2} \in (800, 1200)$ GeV.

### $(m_{0}, A_0)$ Planes

Figure 6 displays some $(m_{0}, A_0)$ planes for fixed $(\tan \beta , m_{1/2}) = (15, 800/1200)$ GeV in the upper/lower panels, showing the same mass and relic density contours as in the previous figures. The relic density strip now tends to larger $m_\mathrm{H}$ as $m_0$ is increased. In the upper panel, we find LHC-compatible values of $m_\mathrm{H}$ along all of the displayed portion of the relic density contour from $m_{0} \in (2200, 2600)$ GeV, and similarly in the lower panel for $m_{0} \in (2400, 3600)$ GeV.

## Phenomenology along stop coannihilation strips

Having established the context for our study of stop coannihilation strips, we now consider in more detail phenomenological constraints and possible experimental signatures along these strips. In general, the value of $\delta m \equiv m_{\tilde{t}_1} - m_\chi$ plays an important rôle in this phenomenology, falling to zero at the tip of the strip. Typical values of $\delta m$ can be inferred from Figs. 3, 4, 5 and 6, where we see that the $m_\mathrm{H}$-compatible regions of the $\Omega _\chi h^2 = 0.12$ strip generally have $m_\chi + m_c < m_{\tilde{t}_1} < m_\chi + m_b + m_\mathrm{W}$. However, we emphasise that smaller values of $\delta m$ would be allowed if the neutralino LSP provided only a fraction of the astrophysical cold dark matter.

### Strips for fixed $A_0/m_0$

**Fig. 7:**
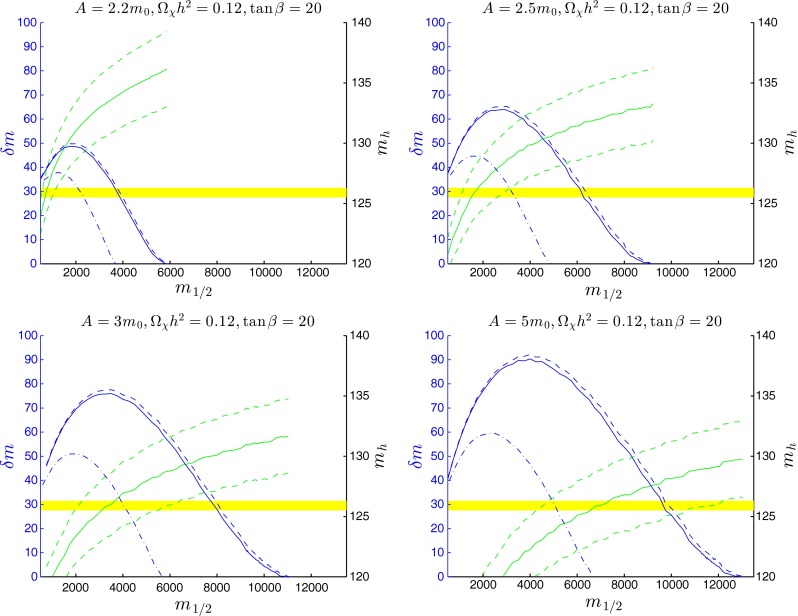
The mass difference $\delta m=m_{\tilde{t}_1}-m_\chi$ and the Higgs mass $m_\mathrm{H}$ (all masses in GeV units) as functions of $m_{1/2}$ along the coannihilation strip where $\Omega _{\chi } h^2=0.12$, for $\tan \beta =20$ and $A=2.2 \, m_{0}, 2.5 \, m_0, 3.0 \, m_0$ and $5.0 \, m_0$. The *solid blue lines* show the values of $\delta m$ incorporating the Sommerfeld corrections. The *dashed blue lines* show $\delta m$ with $\Omega _{\chi } h^2=0.125$ and the *dot-dashed blues line* show $\delta m$ without the Sommerfeld correction. The *green lines* show the values of $m_\mathrm{H}$, with the *dashed lines* representing the uncertainty range given by FeynHiggs 2.10.0

Figure 7 shows $\delta m = m_{\tilde{t}_1}-m_\chi$ and $m_\mathrm{H}$ as functions of $m_{1/2}$ along the coannihilation strip where $\Omega _{\chi }h^2=0.12$, for $\tan \beta = 20$ and $A_0 = 2.2 \, m_{0}, 2.5 \, m_0, 3.0 \, m_0$ and $5.0 \, m_0$. The solid blue lines show the values of $\delta m$ incorporating the Sommerfeld corrections, and the lower dashed blue lines show the values of $\delta m$ that would be required in the absence of the Sommerfeld corrections. The inclusion of the Sommerfeld effects increases significantly $\delta m$ for generic values of $m_{1/2}$, and also extends significantly the length of the stop coannihilation strip. For $A_0 = 2.2 \, m_0$, we see that $\delta m$ rises to a maximum $\sim$50 GeV at $m_{1/2} \sim 2000$ GeV, before falling to zero at $m_{1/2} \sim 6000$ GeV, corresponding to $m_{\tilde{t}_1} = m_\chi \sim 3000$ GeV. However, these values are not universal, with a maximal value of $\delta m > 60$ GeV being attained at $m_{1/2} \sim 3000$ GeV for $A_0 = 2.5 \, m_0$ and the tip of the coannihilation strip increasing to $\sim$9000 GeV, corresponding to $m_{\tilde{t}_1} = m_\chi \sim 4600$ GeV. These values increase further to $\delta m > 75 (90)$ GeV at $m_{1/2} = 3500 (4000)$ GeV with the tip at $m_{1/2} = 11000 (13000)$ GeV for $A_0 = 3 (5) \, m_0$, corresponding to $m_{\tilde{t}_1} = m_\chi \sim 5500 (6500)$ GeV. This non-universality reflects the model-dependence of the ${\tilde{t}_1} - {\tilde{t}_2} - h$ coupling noted in (5). The upper dashed blue lines in Fig. 7 show the values of $\delta m$ that would be required for $\Omega _{\chi }h^2=0.125$, 2$\sigma$ above the central value for $\Omega _{\chi } h^2$. We see that the astrophysical uncertainty in $\Omega _{\chi } h^2$ does not impact significantly the length of the stop coannihilation strip.

The yellow bands in Fig. 7 represent the current measurement of $m_\mathrm{H}$, with its experimental error, and the green lines show the values of $m_\mathrm{H}$ calculated with FeynHiggs 2.10.0, where the dashed lines represent the estimated uncertainty range also determined using FeynHiggs 2.10.0. We note that only parts of the stop coannihilation strips are compatible with the LHC measurement of $m_\mathrm{H}$, even after including the FeynHiggs 2.10.0 uncertainty. For $A_0/m_0 = 2.2$, we are restricted to $m_{1/2} \lesssim 1000$ GeV. The allowed range jumps to $1000 \lesssim m_{1/2} \lesssim 3000$ GeV for $A_0 = 2.5 \, m_0$, to the range $(2000, 6000$ GeV for $A_0 = 3 \, m_0$ and the range $(4000, 12000)$ GeV for $A_0 = 5 \, m_0$.

**Fig. 8:**
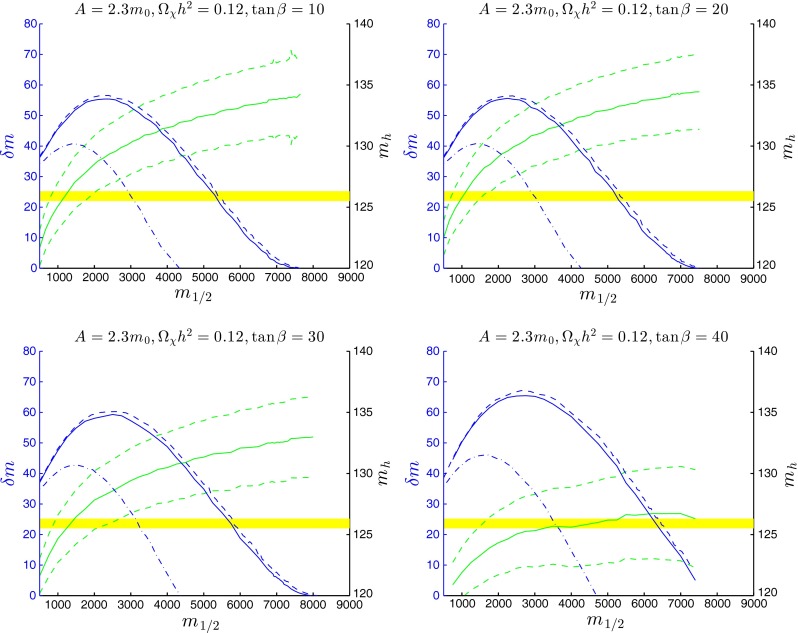
As in Fig. 7, but for $A=2.3 \, m_{0}$ and $\tan \beta =10,20,30$ and $40$

Figure 8 shows the mass difference $\delta m = m_{\tilde{t}_1}-m_\chi$ and $m_\mathrm{H}$ as functions of $m_{1/2}$ along the stop coannihilation strips for $A_0 = 2.3 \, m_0$ and $\tan \beta = 10, 20, 30$ and 40. For this value of $A_0$ the maximum values of $\delta m$ exceed 50 GeV for $\tan \beta = 10, 20$ and 30, and are attained for values of $m_{1/2} \gtrsim 2000$ GeV. For $\tan \beta = 40$, the maximum value of $\delta m$ is above 60 GeV, and it is achieved for $m_{1/2} \sim 3000$ GeV. Correspondingly, the tips of the stop coannihilation strips are not universal, extending from $\sim$7500 GeV for $\tan \beta = 10$ and 20 to $\sim$8000 GeV for $\tan \beta = 30$ and $\sim$8500 GeV for $\tan \beta = 40$. The strips for $\tan \beta = 10$ and $20$ are compatible with $m_\mathrm{H}$ only for $m_{1/2} \lesssim 2000$ GeV, and that for $\tan \beta = 30$ is compatible for $m_{1/2} \lesssim 2500$ GeV, whereas the full coannihilation strip for $\tan \beta = 40$ above 1500 GeV is compatible with $m_\mathrm{H}$ within the theoretical uncertainties.

We display in Table 1 the principal parameters characterizing the end-points of the stop coannihilation strips in the CMSSM for $A_0 = 2.2 \, m_0, 2.5 \, m_0, 3 \, m_0$ and $5 \, m_0$ and $\tan \beta = 20$, and for $A_0 = 2.3 \, m_0$ and $\tan \beta = 10, 20, 30$ and 40, noting their values of $m_0$ and $m_{1/2}$ and the corresponding values of $m_\chi = m_{\tilde{t}_1}$ as well as other parameters that are important for determining the end-points.

**Table 1:** Parameters characterizing the end-points of the stop coannihilation strips in different CMSSM scenarios with fixed $\tan \beta$ and varying $A_0/m_0$ (left columns) and with fixed $A_0/m_0$ and varying $\tan \beta$ (right columns). The values of $m_{1/2}$, $m_0$ and $A_0$ are specified at the GUT scale, whereas the other parameters are specified at the weak scale. Mass parameters are given in GeV and, with the exception of $m_\mathrm{H}$, quoted to 100 GeV accuracy

Parameter	$\tan \beta = 20$				Parameter	$A=2.3\, m_0$			
$A_0/m_0$	$2.2$	$2.5$	$3.0$	$5.0$	$\tan \beta$	$10$	$20$	$30$	$40$
$m_{1/2}$	$5900$	$9200$	$11000$	$13000$	$m_{1/2}$	$7600$	$7500$	$8000$	7600
$m_0$	$24800$	$19400$	$15300$	$8800$	$m_0$	$20900$	$22200$	$26900$	38600
$A_0$	$54600$	$48500$	$45900$	$44200$	$A_0$	$48000$	$51100$	$61900$	88800
$\mu$	$18600$	$18800$	$19400$	$20300$	$\mu$	$18200$	$18500$	$21100$	27000
$A_t$	$25700$	$30100$	$32600$	$35600$	$A_t$	$27300$	$27900$	$31100$	36200
$\sin \alpha$	$-0.060$	$-0.059$	$-0.059$	$-0.059$	$\sin \alpha$	$-0.11$	$-0.059$	$-0.042$	$-$0.034
$m_{\tilde{t}_2}$	$17500$	$16600$	$16200$	$16100$	$m_{\tilde{t}_2}$	$17100$	$16900$	$18100$	20300
$m_\chi = m_{\tilde{t}_1}$	$3000$	$4600$	$5500$	$6500$	$m_\chi = m_{\tilde{t}_1}$	$3800$	$3800$	$4000$	3900
$m_\mathrm{H}$	$136.1$	$133.3$	$131.7$	$129.8$	$m_\mathrm{H}$	$134.2$	$134.5$	$133.1$	126.2

### Strips for fixed $m_0/m_{1/2}$

We have also considered coannihilation strips for fixed values of $m_0/m_{1/2}$ and $\tan \beta$, i.e., rays in the $(m_{1/2}, m_0)$ plane. The values of $A_0/m_0$ are adjusted point-by-point along such lines to obtain the desired value of $\Omega _\chi h^2$.

**Fig. 9:**
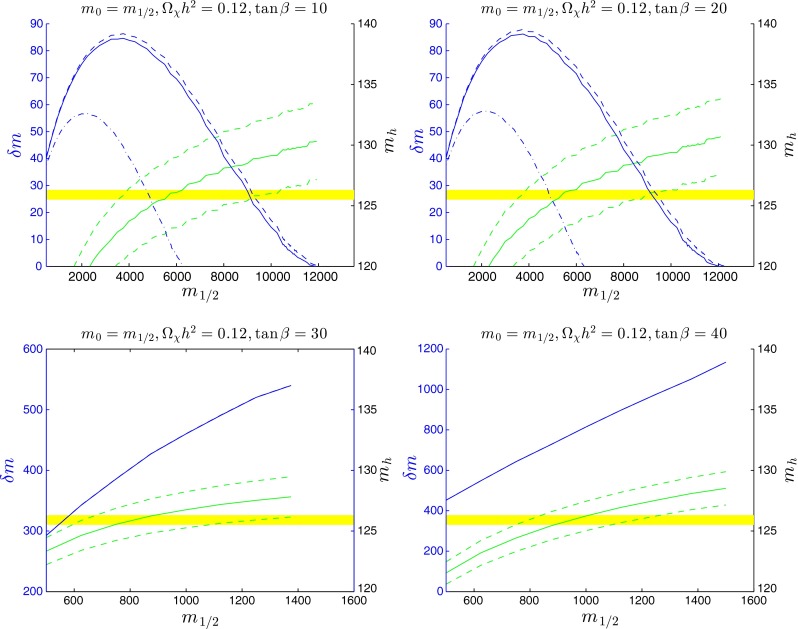
As in Fig. 7, but for $m_{0} = m_{1/2}$ and $\tan \beta =10, 20, 30$ and $40$

Figure 9 shows the behaviours of $\delta m$ and $m_\mathrm{H}$ along coannihilation strips for fixed $m_0 = m_{1/2}$ for the choices $\tan \beta = 10, 20, 30$ and 40. In the upper left panel for $\tan \beta = 10$ we see that $\delta m$ is maximised at $\sim$83 GeV for the nominal value $\Omega _\chi h^2 = 0.120$, when $m_{1/2} \sim 4000$ GeV. This value of $\delta m$ is just below the threshold for ${\tilde{t}_1} \rightarrow \chi + b + W$ decay. The end-point of this strip is at $m_{1/2} \sim 12000$ GeV corresponding to $m_\chi = m_{\tilde{t}_1} \sim 5900$ GeV, and the portion of the strip with $m_{1/2} \in (4000, 10000)$ GeV has a value of $m_\mathrm{H}$ compatible with the LHC measurement within the FeynHiggs 2.10.0 uncertainties. The upper right panel for $\tan \beta = 20$ is quite similar, with $\delta m$ rising slightly higher, but still below $m_\chi + m_\mathrm{W} + m_b$ for $\Omega _\chi h^2 = 0.120$. The lower panels for $\tan \beta = 30$ and 40 are very different. Indeed, in these cases the appropriate relic density is found along the stau coannihilation strip, and the ends of the blue lines in these panels mark the tips of the corresponding stau coannihilation strips. In the $\tan \beta = 30$ case, the whole strip with $m_{1/2} \gtrsim 600$ GeV is compatible with the measured value of $m_\mathrm{H}$, and in the $\tan \beta = 40$ case the portion with $750 \lesssim m_{1/2} \lesssim 1250$ GeV is compatible. However, in both cases the portions with $m_{1/2} \lesssim 800$ GeV are excluded by the ATLAS jets + $/ E_T$ constraint, and the $B_s \rightarrow \mu ^+ \mu ^-$ constraint excludes the portion of the $\tan \beta = 30$ strip with $m_{1/2} \lesssim 100$ GeV and all of the $\tan \beta = 40$ strip.

**Fig. 10:**
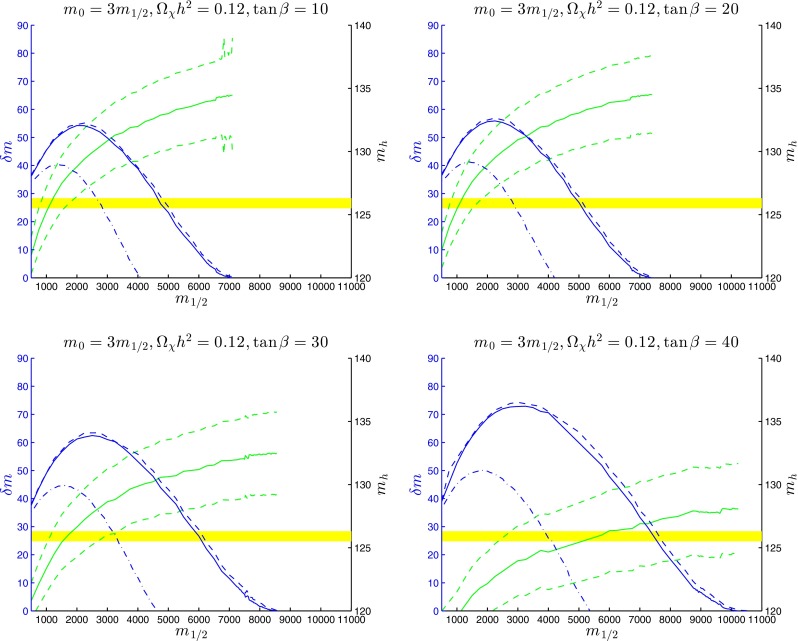
As in Fig. 7, but for $m_{0} = 3 \, m_{1/2}$ and $\tan \beta =10, 20, 30$ and $40$

Figure 10 shows the behaviours of $\delta m$ and $m_\mathrm{H}$ along the corresponding stop coannihilation strips for fixed $m_0 = 3 \, m_{1/2}$ for the choices $\tan \beta = 10, 20, 30$ and 40. In these cases, we see again that the maximum value of $\delta m$ increases with $\tan \beta$ from $\sim 53$ GeV at $m_{1/2} \sim 2000$ GeV when $\tan \beta = 10$ to $\sim$70 GeV at $m_{1/2} \sim 3000$ GeV when $\tan \beta = 40$. Likewise, the tip of the coannihilation strip extends from $\sim$7000 GeV when $\tan \beta = 10$ to $\sim$10000 GeV when $\tan \beta = 40$. In the cases $\tan \beta = 10$ and 20, the calculated value of $m_\mathrm{H}$ is compatible with the value measured at the LHC for $1000 \lesssim m_{1/2} \lesssim 2000$ GeV, rising to $\lesssim$3000 GeV when $\tan \beta = 30$ and the range $\gtrsim$2000 GeV when $\tan \beta = 40$.

Table 2 lists relevant parameters of the end-points of the stop coannihilation strips for $m_0/m_{1/2} = 1$ and $\tan \beta = 10$ and 20, and for $m_0/m_{1/2} = 3$ and $\tan \beta = 10, 20, 30$ and 40.

### Stop decay signatures along the coannihilation strip

We now consider the stop decay signatures along the coannihilation strips discussed in the previous section. Generally speaking, one expects the two-body decays ${\tilde{t}_1} \rightarrow \chi + c$ to dominate as long as $\delta m > m_\mathrm{D} \sim$1.87 GeV [104–106]. Below this threshold, the dominant two-body decay processes are ${\tilde{t}_1} \rightarrow \chi + u$, which would lead to decays of a mesino ${\tilde{t}_1}{\bar{q}} \rightarrow \chi +$ non-strange mesons and of a sbaryon ${\tilde{t}_1} qq \rightarrow \chi +$ baryon, etc. Four-body decays ${\tilde{t}_1} \rightarrow \chi + b + \ell + \nu$ and ${\tilde{t}_1} \rightarrow \chi + b + u + {\bar{d}}$ are also important as long as $\delta m > m_B \sim$5.3 GeV, together with ${\tilde{t}_1} \rightarrow \chi + b + c + {\bar{s}}$ when $\delta m > m_{B_s} + m_\mathrm{D} \sim m_{B} + m_{D_s} \sim m_{B_c} + m_K \sim$7 GeV. Above this threshold, the total four-body decay rate $\sim$9$\Gamma ({\tilde{t}_1} \rightarrow \chi + b + \ell + \nu )$.

**Table 2:** As in Table 1, but for CMSSM scenarios with fixed $m_0/m_{1/2} = 1$ and $3$

Parameter	$m_0/m_{1/2} = 1$		$m_0/m_{1/2} = 3$			
$\tan \beta$	$10$	$20$	$10$	$20$	$30$	$40$
$m_{1/2}$	$11900$	$12100$	$7100$	$7400$	$8600$	$10200$
$m_0$	$11900$	$12100$	$21300$	$22200$	$25700$	$30700$
$A_0$	$43500$	$44700$	$48100$	$50900$	$60000$	$73200$
$\mu$	$19700$	$19800$	$18000$	$18400$	$20900$	$24500$
$A_t$	$33600$	$34100$	$26400$	$27600$	$31600$	$36900$
$\sin \alpha$	$-0.11$	$-0.059$	$-0.11$	$-0.059$	$-0.042$	$-0.033$
$m_{\tilde{t}_2}$	$16500$	$16100$	$17000$	$16800$	$17800$	$18900$
$m_\chi = m_{\tilde{t}_1}$	$5900$	$6000$	$3500$	$3700$	$4300$	$5200$
$m_\mathrm{H}$	$130.3$	$130.7$	$134.5$	$134.6$	$132.7$	$128.6$

**Fig. 11:**
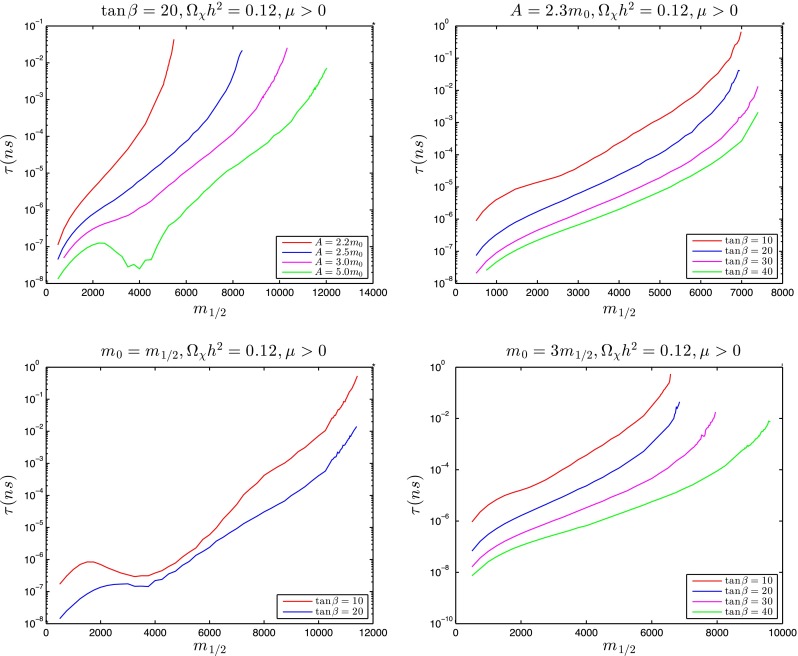
The total ${\tilde{t}_1}$ lifetime along the stop coannihilation strips (*upper left*) for $\tan \beta = 20$ and $A_0 = 2.2 \,m_0$ (*red*), $2.5 \, m_0$ (*blue*), $3.0 \, m_0$ (*purple*) and $5.0 \, m_0$ (*green*) (*upper right*) for $A_0 = 2.3 \, m_0$ when $\tan \beta = 10$ (*red*), $20$ (*blue*), $30$ (*purple*) and $40$ (*green*) (*lower left*) for $m_0 = m_{1/2}$ and $\tan \beta = 10$ (*red*) and $\tan \beta = 20$ (*blue*), and (*lower right*) for $m_0 = 3 \, m_{1/2}$ and $\tan \beta = 10$ (*red*), 20 (*blue*), 30 (*purple*) and 40 (*green*). The *lines* are restricted to the ranges of $m_{1/2}$ where $\delta m > m_\mathrm{D} \sim$1.87 GeV

Figure 11 displays calculations of the total ${\tilde{t}_1}$ lifetime along the stop coannihilation strips for $\tan \beta = 20$ and $A_0 = 2.2 \, m_0, 2.5 \, m_0, 3 \, m_0$ and $5 \, m_0$ (upper left panel), and for $A_0 = 2.3 m_0$ with $\tan \beta = 10, 20, 30$ and 40 (upper right panel), truncated to the ranges where $\delta m > m_\mathrm{D} \sim$1.87 GeV. Our results are, in general, qualitatively consistent with those of previous authors [104–106]. In general, we see that the lifetime $\tau _{\tilde{t}_1}$ increases as $m_{1/2}$ increases monotonically towards the end of the coannihilation strip, reaching $\tau _{\tilde{t}_1} \sim 1$ ns near the end of the strip for $A_0 = 2.3 \, m_0$ and $\tan \beta = 10$.^8^ The lifetime would be further enhanced when $\delta m < m_\mathrm{D}$, by a CKM matrix element factor $\mathcal{O}(20)$ as well as by phase-space suppression, but we do not discuss this possibility in detail. In the lower left panel of Fig. 11 we display the corresponding calculations of the total ${\tilde{t}_1}$ lifetime for the stop coannihilation strips with $m_0 = m_{1/2}$ and $\tan \beta = 10$ and $20$, and in the lower right panel the lifetime along the $m_0 = 3 \, m_0$ strips for $\tan \beta = 10, 20, 30$ and 40. We see that again $\tau _{\tilde{t}_1} \sim 1$ ns near the end of the strip for $m_0 = 3 \, m_{1/2}$ and $\tan \beta = 10$.

**Fig. 12:**
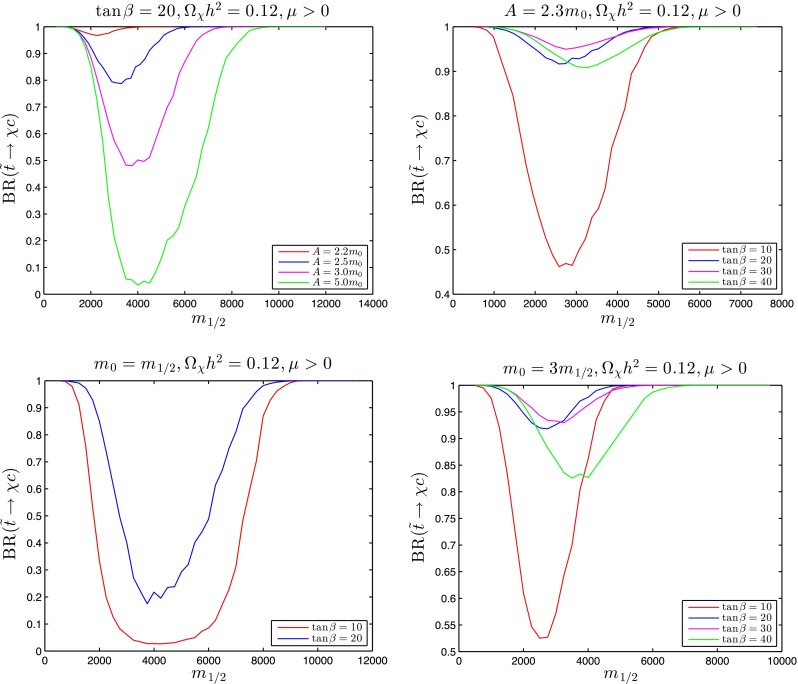
The branching ratios for ${\tilde{t}_1} \rightarrow \chi + c$ decay in the same models as in Fig. 11 and using the *same colours for the lines*

Figure 12 displays calculations of the ${\tilde{t}_1} \rightarrow \chi + c$ branching ratio along the stop coannihilation strips for $\tan \beta = 20$ and $A_0 = 2.2 \, m_0, 2.5 \, m_0, 3 \, m_0$ and $5 \, m_0$ (upper left panel), for $A_0 = 2.3 m_0$ with $\tan \beta = 10, 20, 30$ and 40 (upper right panel) for $m_0 = m_{1/2}$ and $\tan \beta = 10$ and $20$ (lower left panel), and for $m_0 = 3 \, m_0$ and $\tan \beta = 10, 20, 30$ and 40 (lower right panel), again truncated to the ranges where $\delta m > m_\mathrm{D} \sim$1.87 GeV. We see that the two-body decay ${\tilde{t}_1} \rightarrow \chi + c$ is usually more important than the four-body decays ${\tilde{t}_1} \rightarrow \chi + b + f + {\bar{f}'}$, but with important exceptions such as when $\tan \beta = 20, A_0 = 5.0 \, m_0$ for $3000 \lesssim m_{1/2} \lesssim 7000$ GeV and when $m_0 = m_{1/2}$ and $\tan \beta = 20$ for $2000 \lesssim m_{1/2} \lesssim 7500$ GeV. As a general rule, two-body dominance is reduced for intermediate values of $m_{1/2}$ where $\delta m$ is largest and the four-body phase space opens up, in which case four-body decay signatures may become interesting as well as two-body decays. Indeed, for $3000 \lesssim m_{1/2} \lesssim 5000$ GeV when $\tan \beta = 20$ and $A_0 = 5.0 \, m_0$ and when $m_0 = m_{1/2}$ and $\tan \beta = 20$, $\delta m > m_B + m_\mathrm{W}$ so that the three-body decay ${\tilde{t}_1} \rightarrow \chi + b + W$ is formally accessible. In our treatment of this case we calculate ${\tilde{t}_1} \rightarrow \chi + b + (W^* \rightarrow f + {\bar{f}'})$, where $W^*$ denotes an (in general) off-shell $W$ boson represented by a Breit–Wigner line shape. This yields a larger (and more accurate) decay rate than calculating naively the three-body decay to $b$ and an on-shell $W$ boson, and we find that BR(${\tilde{t}_1} \rightarrow \chi + b + f + {\bar{f}'}$) may exceed BR(${\tilde{t}_1} \rightarrow \chi + c$) by over an order of magntitude.

## Summary and conclusions

We have shown in this paper that the existence of a long stop coannihilation strip where the relic neutralino density $\Omega _\chi h^2$ falls within the cosmological range is generic in the CMSSM for $2.2 \, m_0 \lesssim A_0 \lesssim 5.5 \, m_0$. It is essential for calculating the length of this strip and the mass difference $\delta m = m_{\tilde{t}_1} - m_\chi$ along the strip to include Sommerfeld effects. The two annihilation processes that are most important for determining the length of this strip are ${\tilde{t}_1} {\tilde{t}_1}^* \rightarrow$ 2 gluons via t-channel ${\tilde{t}_1}$ exchange and s-channel gluon exchange, which are completely model-independent, and ${\tilde{t}_1} {\tilde{t}_1}^* \rightarrow$ 2 Higgs bosons, which is more model dependent. Specifically, the cross section for the latter process is mediated by ${\tilde{t}_2}$ in the cross channel, and hence it depends on $m_{\tilde{t}_2}$ and on the ${\tilde{t}_1} - {\tilde{t}_2} - h$ coupling $C_{\tilde{t}_1{-}\tilde{t}_2{-}h}$ (5) in the combination $C_{\tilde{t}_1-\tilde{t}_2-h}/m_{\tilde{t}_2}$. We therefore expect that the location of the end-point of the stop coannihilation strip should depend primarily on this ratio.

**Fig. 13:**
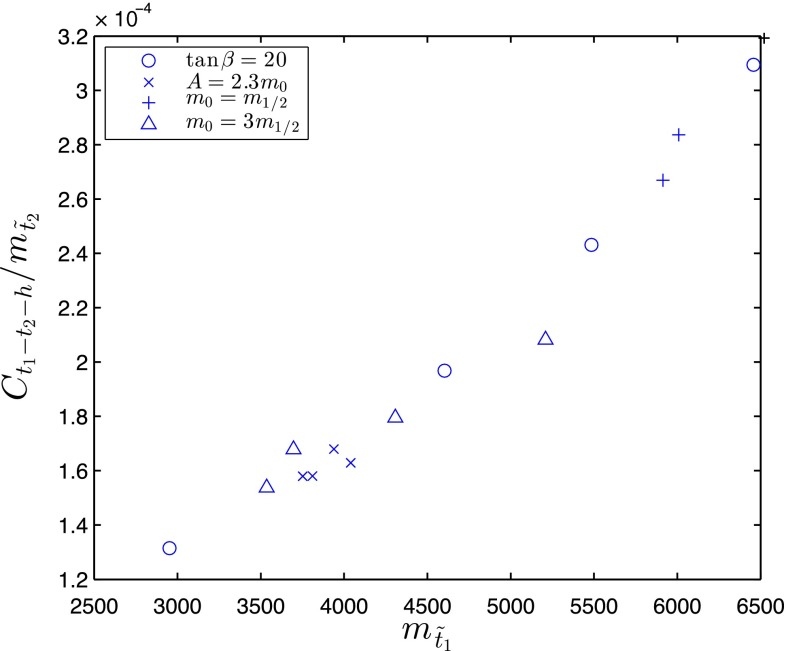
A scatter plot of the end-point values of $m_\chi = m_{\tilde{t}_1}$ vs. the quantity $C_{\tilde{t}_1-\tilde{t}_2-h}/m_{\tilde{t}_2}$ for the models with parameters listed in Tables 1 and 2

In Tables 1 and 2 we have listed the parameters of the end-points in the various cases we have studied, including those appearing in the expression for $C_{\tilde{t}_1-\tilde{t}_2-h}$ (5). In Fig. 13 we display a scatter plot of the end-point values of $m_\chi = m_{\tilde{t}_1}$ vs. the quantity $C_{\tilde{t}_1-\tilde{t}_2-h}/m_{\tilde{t}_2}$. We see that, to a good approximation, the end-point of the stop coannihilation strip is indeed a simple, monotonically increasing function of $C_{\tilde{t}_1-\tilde{t}_2-h}/m_{\tilde{t}_2}$. As seen in Fig. 13, in the models we have studied the maximum value of $m_\chi = m_{\tilde{t}_1}$ compatible with the cosmological dark matter constraint is $\sim$6500 GeV. As seen in the tables, these scenarios yield large values of $m_\mathrm{H}$ as calculated using FeynHiggs 2.10.0, but when $\tan \beta = 40$ the end-points are compatible with the measured value of $m_\mathrm{H}$ within the calculational uncertainty of $\sim$3 GeV. It seems possible that larger values of $m_\chi = m_{\tilde{t}_1}$ would be possible in models with larger values of $C_{\tilde{t}_1-\tilde{t}_2-h}/m_{\tilde{t}_2}$.

We infer that a high-mass end-point for a stop coannihilation strip is likely to be a general feature of a broad class of models. Its appearance is not restricted to the CMSSM and closely related models such as the NUHM [107–112], and its location depends primarily on the combination $C_{\tilde{t}_1-\tilde{t}_2-h}/m_{\tilde{t}_2}$. However, the extent of the stop coannihilation strip might be increased further in models in which other sparticles are (almost) degenerate with the ${\tilde{t}_1}$ and $\chi$. This might occur, for instance, in circumstances under which the lighter sbottom ${\tilde{b}_1}$ or one or more squarks of the first two generations happened to be nearly degenerate with the ${\tilde{t}_1}$ and $\chi$, but this is unlikely to be a generic model feature.

We note also that the dominant ${\tilde{t}_1}$ decay mode along the stop coannihilation strip is likely to be ${\tilde{t}_1} \rightarrow \chi + c$, since the mass difference $\delta m = m_{\tilde{t}_1} - m_\chi < m_B + m_\mathrm{W}$ in general and four-body decays ${\tilde{t}_1} \rightarrow \chi + b + f + \bar{f^\prime }$ are strongly suppressed by phase space. This is likely to be a generic feature of stop coannihilation strips. We also note that the ${\tilde{t}_1}$ lifetime may approach a nanosecond near the tip of the stop coannihilation strip, which is also likely to be a generic feature.

We conclude that the stop coannihilation strip may be distinctive as well as generic.

## Footnotes

1 It is well established that perturbative QCD can be used to describe cross sections for processes involving heavy coloured particles just above their thresholds, cf, the use of perturbative QCD to describe the $\mathrm{e}^+ \mathrm{e}^- \rightarrow {\bar{t}} t$ threshold. Since $m_{\tilde{t}_1} > m_t$ in the region of interest, perturbative QCD will be even more reliable for calculating ${\bar{\tilde{t}}_1} {\tilde{t}_1}$ annihilation close to threshold. Specifically, this is done in [69].

2 We take the opportunity to recall that radiative corrections to stop coannihilation processes have been calculated in [63]. Their effects are, in general, smaller than other uncertainties in our calculations and are not included in our analysis.

3 We have not taken into account the possible role of charge and colour breaking minima here. For a recent study of these effects see [90].

4 The widths of these dark matter strips have been enhanced for visibility. Barely visible in the lower parts of the unshaded wedges between the strips in some panels of Figs. 1 and 2 are low densities of points where annihilations of other sparticles coannihilating with the neutralino are enhanced by direct-channel Higgs poles, reducing $\Omega _\chi h^2$ into the allowed range.

5 The sensitivity of the CMSSM limits to $\tan \beta$ and $A_0$ has been studied in both previous experimental papers and in [96], where it was shown that the limits form jets + MET searches in the region of interest are insensitive to these parameters.

6 For $\tan \beta = 20$ and $A_0 = 5.5 \, m_0$ the neutralino LSP region is reduced to a very narrow slit extending from $(m_{1/2}, m_0) = (500, 400)$ to $(4500, 3000)$ GeV.

7 We note that the ATLAS search for jets + $/ E_T$ events, the measurement by CMS and LHCb of $B_s \rightarrow \mu ^+ \mu ^-$ decay and the experimental constraint on $b \rightarrow s \gamma$ do not constrain any of the strip regions shown in Figs. 3, 4, 5 and 6.

8 Exceptions are seen in the left panels of Fig. 11. The dips in the lifetime arise because $\delta m \sim m_B + m_\mathrm{W}$, as seen in the lower right panel of Fig. 7 and the upper panels of Fig. 9.
